# Quantitative DNA Analyses for Airborne Birch Pollen

**DOI:** 10.1371/journal.pone.0140949

**Published:** 2015-10-22

**Authors:** Isabell Müller-Germann, Bernhard Vogel, Heike Vogel, Andreas Pauling, Janine Fröhlich-Nowoisky, Ulrich Pöschl, Viviane R. Després

**Affiliations:** 1 Biogeochemistry and Multiphase Chemistry Departments, Max Planck Institute for Chemistry, Mainz, Germany; 2 Geosciences, Johannes Gutenberg University, Mainz, Germany; 3 Institute for Meteorology and Climate Research, Karlsruhe Institute of Technology (KIT), Eggenstein-Leopoldshafen, Germany; 4 MeteoSchweiz, Zürich-Airport, Zürich, Switzerland; 5 Department of General Botany, Johannes Gutenberg University, Mainz, Germany; Peking University, CHINA

## Abstract

Birch trees produce large amounts of highly allergenic pollen grains that are distributed by wind and impact human health by causing seasonal hay fever, pollen-related asthma, and other allergic diseases. Traditionally, pollen forecasts are based on conventional microscopic counting techniques that are labor-intensive and limited in the reliable identification of species. Molecular biological techniques provide an alternative approach that is less labor-intensive and enables identification of any species by its genetic fingerprint. A particularly promising method is quantitative Real-Time polymerase chain reaction (qPCR), which can be used to determine the number of DNA copies and thus pollen grains in air filter samples. During the birch pollination season in 2010 in Mainz, Germany, we collected air filter samples of fine (<3 μm) and coarse air particulate matter. These were analyzed by qPCR using two different primer pairs: one for a single-copy gene (*BP8*) and the other for a multi-copy gene (ITS). The *BP8* gene was better suitable for reliable qPCR results, and the qPCR results obtained for coarse particulate matter were well correlated with the birch pollen forecasting results of the regional air quality model COSMO-ART. As expected due to the size of birch pollen grains (~23 μm), the concentration of DNA in fine particulate matter was lower than in the coarse particle fraction. For the ITS region the factor was 64, while for the single-copy gene *BP8* only 51. The possible presence of so-called sub-pollen particles in the fine particle fraction is, however, interesting even in low concentrations. These particles are known to be highly allergenic, reach deep into airways and cause often severe health problems. In conclusion, the results of this exploratory study open up the possibility of predicting and quantifying the pollen concentration in the atmosphere more precisely in the future.

## Introduction

Pollen allergies affect up to 30% of the human population in industrialized countries [1] and are today the most common form of seasonal respiratory diseases in Europe [2,3]. Although much research has been done to reduce allergic reactions, the most effective way is still the complete avoidance of pollen and their allergens [4]. The establishment and enhancement of accurate pollen quantification and forecasting systems are thus essential for the improvement of public health.

Currently, pollen forecasts are mainly based on the results provided by microscopic analyses of pollen traps, in which pollen and spores are collected [5] and identified based on optical recognition of specific morphological features [6]. Pollen identification thus relies on the precise knowledge of each individual pollen-counting person. Pollen forecasts are based on weather forecasts and the described pollen identification data, which often only comes with a time delay of one or two days. This method is still the standard for national pollen networks covering most of the European continent [1,5].

However, the errors in this pollen counting procedure range from 10% to over 30% [7] depending on pollen abundance, sample size, and the experience of the pollen-counting person. The pollen measurement network in Germany analyses sub-samples of 24 h pollen samples [3]. Thus, only a small proportion of the available pollen material is provided to the forecast system. In addition, species specific pollen determination under a light microscope is challenging as pollen grains from the same genus or belonging to the same family usually cannot be distinguished as they share the same morphologic features [8].

Hence, the improvement of the actual pollen forecasting system is important to help people suffering from pollinosis, especially as the number of sufferers is continuously increasing ([2] and references therein). During the last decade, sophisticated numerical models were developed to simulate the distribution of pollen, based on the experience gained with the simulation of other aerosol particles like soot, mineral dust, sea salt, and particles formed from gaseous precursors. Gaussian types of models can only be applied very locally and they are not suitable for larger areas [9,10]. Other models can be used for larger areas and for species specific pollen [11–15]. In this study we applied the fully online coupled model system COSMO-ART [16,17] to simulate the temporal and spatial distribution of *Betula* spp. (birch) pollen.

A major bottleneck for the COSMO-ART model and all the other numerical dispersion models, however, are the uncertainties of the available pollen data. Reliable measurements of species specific pollen concentrations while being aerosolized are necessary for two purposes when numerical pollen forecasts are performed. The data are needed to validate the model results which are then used to derive optimally tuned parameters for the various emission processes (e.g., dependence of emission on temperature and humidity, drying of the plants after precipitation, plant-related processes).

One possible solution is the molecular biological quantification of airborne allergenic plant and pollen material. Molecular methods can often be automated and applied faster and are—when established—less error prone. With species specific primer pairs individual species of interest can be quantified and provide better estimates of the pollen concentrations.

Birch is well-known as the most important and recognized aero-allergenic trees in Central and North-Western Europe [18–21]. It has an important impact on human health by causing seasonal hay fever, pollen-related asthma [20], and other health problems such as cross-reactive food allergy, called oral food syndrome [22–24]. Furthermore, allergen-inducing ingredients appear to be present in plant parts other than pollen for birch [25]. Thus, fragments of plant material are also important from an allergological point of view. They are morphologically unidentifiable by microscopic examination [26] but detectable with molecular biological methods.

The aim of the present exploratory study is, to test and establish quantitative Real-Time PCR (qPCR) as a new method to quantify airborne plant material. The quantity of DNA originating from *Betula pendula* in the air was measured by qPCR and compared to pollen number concentrations calculated with COSMO-ART for the birch pollen season 2010. In addition, we compared the results to available conventional pollen reference measurements.

Two different DNA regions were used for the quantification of birch DNA extracted from air filter samples: The single-copy *BP8* gene encodes a putative 53 kDa protein that belongs to the group of inducible late embryogenesis abundant proteins [27] and allows species-specific (*B*. *pendula*) DNA identification. For a comparison the well-known multi-copy ITS gene was also used, which is often used for reconstructing plant genera phylogenies ([28] and references therein). As the ITS region only allows genus-specific (*Betula* spp.) DNA quantification, it can be used for comparisons to pollen counts which are also not based on species specific data.

The positive quantification results of birch DNA seem to originate primarily from airborne pollen material and were well-comparable to simulated data of the model system COSMO-ART.

## Materials and Methods

### Aerosol sampling

Aerosol samples were collected on glass fiber filters (Pall Corporation, Type A/A, 102 mm diameter (see also [29]). The sampling station was positioned on a mast at the top of the Max Planck Institute for Chemistry (MPIC, about 5 m above the flat roof of the 3-story building; ~20 m above ground level) on the campus of the University of Mainz (49°59’31.36”N 8°14’15.22”E). The air masses sampled at the MPIC represent a mixture of urban and rural continental boundary layer air in central Europe. A high-volume dichotomous sampler (self-constructed based on Solomon et al., 1983 [30] was used to separate and collect coarse and fine aerosol particles on a pair of glass fiber filters. The sampler was operated with a rotary vane pump (Becker VT 4.25) at a total flow rate of ~300 l min^-1^, corresponding to a nominal cut-off diameter of ~3 μm. Coarse particles with aerodynamic diameters larger than the cut-off were collected through a virtual impactor operated in line with the inlet (~30 l min^-1^), and fine particles with aerodynamic diameters smaller than the cut-off were collected from the main gas flow perpendicular to the inlet (~270 l min^-1^). While 10% of the fine particles are collected on the coarse particle filter as a result of the airflow design of the virtual impactor, the fine particle sample is essentially free from coarse particle contamination [30]. Samples were originally sampled for a biodiversity study of airborne plant material and thus the sampling period was generally ~7 days, corresponding to a sampled air volume of ~3000 m^3^. To ensure contamination free bioaerosol collection, before sampling, all glass fiber filters were baked at 500°C overnight as described in detail by Després et al., 2007 [31]. Loaded filters were packed in aluminum foil (also prebaked at 500°C), and stored in a freezer at -80°C until DNA extraction.

Twelve coarse and twelve fine particle filters were collected during the plants’ specific pollination season in 2010 from the middle of March until the start of June in accordance with the time frame used for the model simulations.

### DNA Extraction

For primer and method testing, DNA from 50 mg *Betula pendula* leaf material was extracted with a commercial soil DNA extraction kit (LysingMatrixE, Fast DNA Spin Kit for Soil, MP Biomedicals) according to the supplier’s instructions. This tested method was then used to extract DNA from air filter sample aliquots (⅛). To monitor the extraction process and control for a contamination free procedure, extraction and filter blanks were always done in parallel. During the DNA extraction process DNA can be lost. The Fast DNA Spin Kit for Soil used in this study, produced the smallest amounts of lost DNA in an intercomparison with other kits as discussed in Mumy and Findlay, 2004 [32]. However, the exact extraction efficiency true for birch pollen grains cannot be deduced from this paper but would need to be measured in a follow-up study.

### Primer Design

Genomic regions commonly used for taxonomic identification in the domain Eukarya (animals, plants, and fungi) are the ribosomal RNA (rRNA) genes, with their highly variable internal transcribed spacer (ITS) region [33–36]. The primer pair for the multi-copy ITS region was constructed with the free online tool Primer 3.0 [37]. The annealing temperature was determined by a temperature gradient PCR. The forward primer targeting the ITS region of *Betula pendula* is located within the ITS 1 region, whereas the reverse primer is located in the ITS 2 region. Although this ~294 (base pair) bp region is quite variable in copy number and length, even among members of the same population of a single species [38], primer attachment does not cause any problems due to highly conserved sites flanking the ITS region [39].

A *Betula pendula* specific primer pair called *BP8* forward and reverse [6], which amplifies 110 bp of a single-copy gene, was used as well. The sequence and annealing temperature of both primer pairs are listed in Table 1, respectively. As further measurements to control the data quality, the sensitivity and specificity of both primer pairs used in the qPCR was first checked by test PCRs and sequence analysis of ten PCR products as described in detail below.

**Table 1:** Primer pair information.

Primer name	Primer location	Sequence 5’- 3’	*T*_*m*_ [°C]
*Betula* spp. ITS for	ITS	CGG TAG GGA GAC ACT TGT GC	56
*Betula* spp. ITS rev	ITS	GTC CCT TTG CAA GGA GAT GG	56
*BP8* for	*BP8*	ACG ATC GAG TTT TCA TCA AAC AAA	60
*BP8* rev	*BP8*	GAC CTT ATT GTC TTC ACG GTC CTT	60

Primer names, sequences, and specific annealing temperatures [°C] are given. Primer pair ITS *Betula pendula* is self-designed to amplify a multi-copy region, primer pair *BP8* after Longhi et al. 2009 amplifies a single-copy gene [6].

### DNA amplification and cloning

The PCR reactions for the filter samples and positive control reactions were done in a 25 μl mixture with 1 μl template DNA, 1×PCR buffer, 0.2 mM each dNTP (Roth), 0.33 μM of each primer (Sigma-Aldrich), and 2.5 units of JumpStart^TM^ RED*Taq* DNA polymerase (Sigma-Aldrich). To the negative PCR control samples that monitor contaminations, water was added instead of the template DNA. The thermal profile (DNA-Engine PTC 200, Biorad) was as follows: Initial denaturing step at 94°C for 3 min, 35 cycles with denaturing at 94°C for 30 s, annealing at primer pair specific temperature for 30 s (see Table 1), and elongation at 72°C for 90 s; final extension step at 72°C for 5 min. PCR products were separated by electrophoresis (80 V) on a 1% agarose gel and visualized by ethidium bromide staining. The gels were documented with the Gel Doc XR system and analyzed with Quantity One software Version 3.1 (Bio-Rad Laboratories). The DNA size was determined via Molecular Weight Standards (Gene Ruler™ 1 kb DNA Ladder, Fermentas). Primer specificity was monitored by cloning using the TOPO TA Cloning® Kit (Invitrogen) following the supplier`s instruction. Colonies containing inserts were identified by blue-white selection and lysed in 20 μl water for 10 min at 95°C. The inserts were amplified (“Colony PCR”) using 1.5 μl lysate as DNA template. The 25 μl PCR reaction mixture always contained: 12.5 μl JumpStart REDTaq ReadyMix (Sigma-Aldrich) and 0.25 μM of each primer (Sigma-Aldrich). PCR reactions were performed with the primer pair M13F-40 and M13R. The thermal profile for the Colony PCR was as follows: Initial denaturing step at 94°C for 5 min; 40 cycles with 94°C for 30 s, annealing at 55°C for 1 min, elongation at 72°C for 1 min, and final extension step at 72°C for 15 min. First amplification tests were conducted with both primer pairs, ITS and *BP8*, and ten selected PCR products were cloned and sequenced. As only sequences longer than 200 bp are accepted in the GenBank only three of the ITS sequences were deposited with the accession numbers KM102747-KM102749.

### DNA Sequence analysis

DNA sequences were determined with ABI Prism 377, 3100, and 3730 sequencers (Applied Biosystems, Darmstadt) using BigDye-terminator v3.1 chemistry at the DNA Core Facility of the Max Planck Genome Centre Cologne. The sequences were aligned using the program BioEdit [40]. For identification through similarity comparison with known sequences, databank queries using the Basic Local Alignment Search Tool (BLAST) were performed via the website of the National Center for Biotechnology Information (NCBI). All queried sequences returned database hits with 100% identity for *Betula pendula* for both ITS and *BP8* primer pairs. However, for the ITS primer pair sequences of other *Betula spp*. were identified with Blast hits ≤ 99%, thus this primer pair could only be used for genus-specific birch DNA detection.

### Quantitative Real-Time PCR (qPCR)

Quantitative real-time PCR has potential to develop into a useful alternative to conventional pollen counting methods as initial back-of-envelope estimates suggest the qPCR method is ~ 5 times as fast as the traditional microscopic technique (data not shown). Quantitative Real-Time PCR was conducted to measure the amount of *Betula pendula* DNA in continental boundary layer air. The Real-Time PCR MiniOpticon™ System for Real-Time PCR Detection (Biorad) was used as the operation system. Opticon Monitor™ Software (Version 3.1) controls all operations in the Mini Opticon System. Experimental set-up and programming of the qPCR runs followed the supplier’s instructions. The PCR Mix for the qPCR was as follows: 25 μl iQ™ SYBR® Green Supermix, with 1x final concentration, each Primer 0.3 μM, 2 μl DNA template filled with sterile water to the total volume of 50 μl per sample. The PCR protocol was as follows: Initial denaturation step at 95°C for 5 min, followed by the 39 cycles with: denaturation at 95°C for 30 s, annealing at the primer pair specific temperature for 45 s (Table 1), and elongation at 72°C for 1 min. After each elongation a plate read step is conducted, during which the fluorescence of the blank, standard, and sample wells are measured. At the end of the protocol a melting profile is created by raising the temperature from 50°C to 90°C. The fluorescence is measured 1 s after every 0.5°C temperature increase to identify and determine the purity of reaction products.

### Standard curves and preparation

The absolute quantification used in this study is based on comparative measurements with a well-defined standard to determine the absolute amount of the target nucleic acid. During the qPCR run, a standard curve (plot of c(t) value/crossing point against log of amount of standard) of the external standard dilutions is generated and used to determine the copy number of unknown target samples.

*B*. *pendula* DNA was extracted from leaf material, amplified, and cloned as described above. From colonies with inserts that were previously checked by sequence analysis, four *E*.*coli* (Top10, Invitrogen) pre-cultures were prepared as follows: The selected colonies were transferred in 2 ml LB medium (lysogeny broth*)* and 2 μl ampicillin (100 mg ml^-1^) and the pre-cultures were slightly mixed over night at 37°C. Subsequently, a PCR reaction of lysates of the pre-cultures was performed, to check if the vector contained the correct DNA sequence following the protocol of a Colony PCR. To confirm the insertion of the correct DNA fragment a restriction fragment length polymorphism analysis was performed as follows: Two μl of the PCR-products were digested with 5 units of the enzyme *Taq*I (Fermentas). Restriction fragments were separated by gel electrophoresis in a 3% agarose gel and the DNA size was determined via GeneRuler™ Low Range (Fermentas). Restriction fragment patterns were compared to theoretical restriction fragments of the appropriate sequences calculated by pDraw 32 [41].

Afterwards each culture was prepared with 45 ml LB medium, 45 μl ampicillin and 100 μl of the individual pre-cultures and was incubated over night at 37°C. For plasmid preparation of DNA from the recombinant *E*.*coli* cultures, the GenElute™ Plasmid Miniprep Kit (Sigma-Aldrich) was used following the supplier’s instructions.

The concentration of the plasmid DNA was measured with Bio-Rad SmartSpec 3000 UV/Vis spectrophotometer using deionized water (dH_2_O) as a control. DNA extracts were diluted 1:10 and measurements were repeated at least four times for each plasmid DNA extract. Only plasmid DNA extracts that had a ratio of A260/A280 between 1.8 and 2.0 were chosen for the standard preparation. As one plasmid carries exactly one PCR product (either ITS or *BP8*) which can be quantified in the standard, the number of plasmids present in the plasmid DNA extract is essential. To calculate this standard plasmid number (*CopyNo*_*Plasmid*_) per μl we followed the Eq 1 using the measured plasmid DNA extract concentration (*C*_*Plasmid*_, gμl^-1^) as well as the known length (*L*_*Plasmid*_) and weight (*W*_*Plasmid*_, gmol^-1^) of a single plasmid including its inserted PCR product. Being a double strand DNA, the weight of the single plasmid was calculated using the number of base pairs of the plasmid the inserted PCR product (*L*_*Plasmid*_) and twice the average nucleotide mass (330 gmol^-1^) following Lee et al., 2006, Whelan et al., 2003 [42,43]; see Table 2 for symbol explanation. For the standard preparation each plasmid DNA extract was then diluted in eight steps from 10^8^ to 10^1^ copies μl^-1^.

$${\text{CopyNo}}_{\text{Plasmid}}({\mu \text{l}}^{-1})=\frac{{\text{N}}_{\text{A}}({\text{mol}}^{-1})\times {\text{C}}_{\text{Plasmid}}({\text{g}\;\mu \text{l}}^{-1})}{{\text{L}}_{\text{Plasmid}}\times {\text{W}}_{\text{Plasmid}}({\text{gmol}}^{-1})}=\frac{6.02\times 10^{23}({\text{mol}}^{-1})\times {\text{C}}_{\text{Plasmid}}({\text{g}\;\mu \text{l}}^{-1})}{{\text{L}}_{\text{Plasmid}}\times 660\;({\text{gmol}}^{-1})}$$ (1)

**Table 2:** Equation parameter.

Symbol	Quantity
*CopyNo*_*Plasmid*_	Copy number of the standard plasmid per μl extract
*C*_*Plasmid*_	Concentration of the plasmid (including PCR product, g μl^-1^)
*C*_*tot*_	DNA concentration total (number of copies per cubic meter, cp m^-3^)
*C*_*c*_	DNA concentration coarse (cp m^-3^)
*C*_*f*_	DNA concentration fine (cp m^-3^)
*L*_*Plasmid*_	Length of plasmid DNA and included PCR product in base pairs (bp)
*W*_*Plasmid*_	Weight ofplasmid and included PCR product in g mol^-1^
*N*_*A*_	Avogadro constant (mol^-1^)
*N*_*c*_	Number of DNA copies (cp), coarse
*N*_*f*_	Number of DNA copies (cp), fine
*N*_*tot*_	Number of DNA copies (cp), total
*V*_*c*_	Sampled air for coarse flow (m^3^)
*V*_*f*_	Sampled air for fine flow (m^3^)
*V*_*tot*_	Total air flow (m^3^)

Parameters used in Eqs 1–3 are given with their abbreviation, their definition and units.

In these experiments, specific PCRs were pursued to measure the original copy number of a target nucleic acid. This was done by measuring the PCR product concentration and comparing it to the calculated copy numbers of a given concentration of the standard. The copy numbers of the PCR product of interest was derived, based on comparisons with the standard, for 1000 l air (cp m^-3^). Thus, when discussing copy numbers of PCR products throughout this study, the number of measured copies of the PCR product within 1 m^3^ of sampled air is intended. In each qPCR run, the standard was measured three times and each sample two times.

For the absolute quantification of birch DNA in the filter samples, two standards were created, one standard in the multi-copy ITS region and one in the single-copy *BP8* gene, respectively.

### Data Analysis

The quality of a qPCR can be analyzed by both, the *R* ^*2*^ value of the standard as well as the comparability of the results of double or triplet replicates and additionally by the PCR efficiency (*E*), which indicates how much of the template was amplified per cycle [44,45]. Quantitative PCR runs with PCR efficiencies less than 80% were not used for further analysis. For each double assay, the average initial quantity of the template (DNA copies: *N*_*x*_) and the number of DNA copies per m^3^ of sampled air (*C*_*x*_) of a total flow rate during the sampling time of each filter were calculated: For coarse *V*_*c*_ and *C*_*c*_), for fine *V*_*f*_ and *C*_*f*_, and for the total particle size *V*_*tot*_ and *C*_*tot*_ (Table 2 for symbol explanation). Calculations are given in Eqs 2 and 3.

Cc=Nc−Cf⋅VVtot=Nc−Nf⋅VcVfVtot (2)

$$C_{f}=\frac{N_{f}}{V_{f}}$$ (3)

The DNA copy numbers for coarse and fine particle filters were calculated separately as 10% of the fine particles were sampled with the coarse particle filters as a result of the air flow design of the virtual impactor. Eq 2 includes a correction for this fact. However, the fine particle samples are essentially free from coarse particle contamination [30]. Table 2 lists equation parameters for DNA quantification.

Intact pollen grains are only collected on coarse filter samples. Pollen grains contain a haploid vegetative cell and a haploid generative cell. For *B*. *pendula*, the latter cell divides with mitoses into two haploid sperm cells while being airborne [46]. Thus, each pollen grain of *B*. *pendula* contains three haploid genomes and is called tricellular [46]. To estimate the real pollen grain number as exactly as possible, the DNA copy numbers of the coarse fraction were divided by three. Little is known about the proportion of the plant fragments aerosolized alongside the pollen grains nor their deposit rate [47,48]. But as so little is known and the proportion seemingly can be very low, they were disregarded for this first DNA quantification-based pollen estimation.

### Statistical analysis

To investigate possible correlations between the qPCR results, the model simulations for Mainz and Löwenstein, and the pollen trap data, the four different data sets were first tested if they were normally distributed by using the Shapiro-Wilk-Test [49]. To test for the correlation within normally distributed data the Pearson’s linear regression coefficient was calculated using the R package by comparing the measurements and predictions from the same sampling periods [50]. Results are given in the discussion section below.

### Blanks

To monitor contamination during amplification and DNA extraction, each qPCR run included one negative control. Extraction and filter blanks were extracted and amplified along with sampled filters and analyzed in the qPCR. To detect possible contaminations from the sampler and sample handling, blank samples were taken at regular intervals (~4 weeks). The filters were mounted in the sampler as for regular sampling, but the pump was either not turned on at all (“mounting blanks”) or only for 5 s (“start-up blank”). Each blank consists of one filter collecting coarse particles (labeled “a”) and one filter collecting fine particles (labeled “b”). Two mounting and two start-up blanks were analyzed: MZ 327 (start-up blank) and MZ 328 (mounting blank) on 2010-04-06; MZ 333 (start-up blank) and MZ 334 (mounting blank) on 2010-05-04. DNA was not detectable on PCR blanks and extraction blanks. However, in one start-up blank (MZ 333a) birch DNA was detected for the *BP8* gene. With the ITS primer pair, DNA was detected on the start-up blank (MZ 333a) and mounting blanks (MZ 334a and b). All four blank samples were from the plant specific main-pollen season but the PCR efficiency was in all cases below 80% and thus excluded from further analysis.

### Model simulations

The model system COSMO-ART [16] is based on the numerical weather forecast model COSMO (Consortium for Small-scale Modelling; [51]). The extension ART (Aerosols and Reactive Trace Gases) allows the forecast of a variety of aerosol particles including pollen.

The model system includes a parameterization of pollen emission described in Helbig et al., 2004 [52]. Additionally, the model describes all stages of the pollen cycle: start, course and end of the blooming season, advection, turbulent diffusion, wash out, and deposition. The exact knowledge of the number and position of trees and plants is an important input parameter.

In order to test COSMO-ART, the parameterization following Helbig et al., 2004 [52] was adopted for birch pollen and the model results were compared with observations in Switzerland [17]. Estimates of the emission flux of birch pollen were made using a detailed map of the distribution of birch trees within the model domain. The model results were compared with the measured diurnal cycles of the pollen grain concentrations of six pollen counting stations (Burkard traps).

A further improvement of the model system was a detailed map of the distribution of birches over Europe based on a combination of land use data and observed pollen concentrations [53]. Very recently, the model system was extended by the treatment of the dispersion of *Ambrosia* pollen [4] and by a new pollen emission scheme [54]. Since 2011 the model system is operationally used for birch pollen over south and central Europe by Meteoswiss.

In this study birch pollen simulations with COSMO-ART were carried out for a model domain covering Germany with a spatial resolution of 7 x 7 km^2^ for a time period of one month (April, 2010). The model output is available with a time resolution of one hour. The simulated pollen concentrations at the relevant grid point were summed up and averaged to daily mean values to match the exact filter sampling periods. Comparison results are given below.

### Conventional pollen measurements with a Burkard sampler at Löwenstein

As in this exploratory study pre-existing air filter samples were used, no retroactive comparison could be made with the Burkard sampling method. To still be able to compare the methods, publicly available data of average daily birch pollen concentrations per m^3^ air were acquired from the Stiftung Deutscher Polleninformationsdienst (PID). These concentrations were measured via conventional Burkard sampling systems combined with manual microscopic pollen detection. These pollen count data were taken from the nearest available pollen measurement station in Löwenstein (49°5’48.1632”N 9°22’45.1416”E), Baden-Württemberg about 130 kilometers south-east of Mainz during the measurement period from mid-March to mid-June 2010. Pollen data from nearer PID stations, e.g., in Heidelberg, were not available during the qPCR measurement period. For the comparative analysis, hourly average pollen concentrations were adapted to the weekly measurement interval used for pollen quantification via qPCR.

## Results and Discussion

### Comparison of pollen and birch DNA presence

Table 3 lists the quantification results for the 12 analyzed coarse and fine particle filter samples as well as the 4-week simulation results of the COSMO-ART model at Mainz and at Löwenstein and the pollen trap data at Löwenstein. While the DNA on all coarse and fine particle filter samples could be quantified with the ITS primer pair, only ~90% of the coarse and ~45% of the fine particle filters were quantifiable with the *BP8* primer pair. One reason for this result is likely the multi-copy nature of the ITS region, which was easier to amplify and quantify than the single-copy gene *BP8*. However, the ITS primer pair is also not species- but genus-specific and can bind to birch DNA other than *B*. *pendula*. Close to the sampling site only very few trees from other *Betula* species are known to exist. *B*. *nana* is rare in general. The few occurrences in Germany are mainly from Northern and Eastern Germany [55] and some in south close to the Alps. *B*. *pubescens* is spread more evenly in Germany, though not directly in the sampling site area [56]. However, in comparison to *Betula pendula* the occurrence is very small, and thus, although both species can be amplified by the ITS primer pair, likely negligible. In addition to these naturally occurring *Betula* species, there are a number of species that can be obtained in garden centers or tree nurseries. However, as these species are only represented by individual trees in private gardens, they are unlikely to have a significant impact on the results presented in this study.

**Table 3:** Comparison of qPCR results with simulation and pollen trap data.

Filter ID	Sampling period (2010)	ITS, cp m^-3^		*BP8*, cp m^-3^		Simulated pollen concentration in, pollen m^-3^ at		Observed pollen concentration in pollen m^-3^
		coarse	fine	coarse	fine	Mainz	Löwenstein	Löwenstein
MZ 324	2010-03-16 / 2010-03-23	35	1	0	0	n.a.	n.a.	0
MZ 325	2010-03-23 / 2010-03-30	37	55	1	0	n.a.	n.a.	0
MZ 326	2010-03-30 / 2010-04-06	396	3	9	2	85	67	0
MZ 329	2010-04-06 / 2010-04-13	243384	4630	879	24	405	309	98
MZ 330	2010–04013 / 2010-04-20	76207	255	809	5	649	389	173
MZ 331	2010-04-20 / 2010-04-27	19826	427	69	3	124	283	158
MZ 332	2010-04-27 / 2010-05-04	2203	24	71	0	n.a.	n.a	17
MZ 335	2010-05-04 / 2010-05-11	217	11	17	0	n.a.	n.a.	1
MZ 336	2010-05-11 / 2010-05-18	185	13	10	8	n.a.	n.a.	0
MZ 337	2010-05-18 / 2010-05-25	335	12	4	0	n.a.	n.a.	0
MZ 338	2010-05-25 / 2010-06-01	16	5	16	0	n.a.	n.a.	0
MZ 339	2010-06-01 / 2010-060-08	144	50	4	0	n.a.	n.a.	0

DNA concentrations (cp m^-3^) are listed for ITS multi-copy region and *BP8* single-copy gene for coarse and fine particle filter samples, respectively. The original measured DNA copies in the coarse particle fractions were divided by three to correct for the tricellular nature of *Betula pendula* pollen grains. Calculated pollen concentrations with COSMO-ART for Mainz and Löwenstein and measured pollen concentrations in pollen grains m^-3^ via Burkard sampler in Löwenstein were averaged corresponding to the sampling interval of the appropriate filter sample; n.a.: not available.

### Temporal and quantitative comparison of the genes ITS and *BP8*

**Fig 1:**
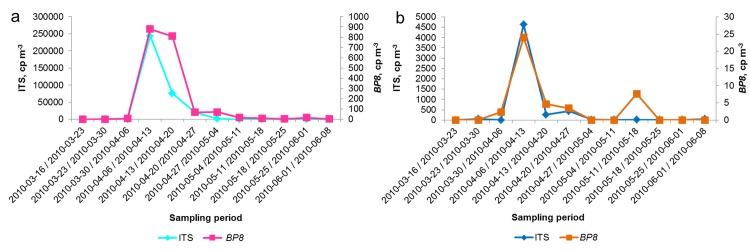
DNA quantification via qPCR measurements. DNA concentrations in copies m^-3^ (cp m^-3^) are given for the multi-copy ITS region and single-copy *BP8* gene for coarse (a) and fine particle filter samples (b) DNA copies m^-3^ are given for ITS quantification on the primary vertical axis, and on the secondary vertical axis for the *BP8* gene. The x-axis shows the time, 2010-03-16 / 2010–0608, during the birch-specific pollination period.

Fig 1A shows, that both the multi-copy as well as the single-copy gene could be quantified in the *B*. *pendula* flowering season between middle of March until the beginning of June. The highest DNA concentrations were measured for the ITS and *BP8* genes during the period of 2010-04-06 to 2010-04-13 in the coarse particulate matter. This time frame corresponds to the main-pollination time of birch in the sampling area. The *BP8* gene experiences another smaller peak in the following week (2010-04-13 / 2010-04-20). The ITS gene copy number shows the same trend though the value is reduced even more compared to the week before.

The temporal progress for the qPCR results in fine particulate matter is illustrated in Fig 1B. Both primer pairs simultaneously show a peak in the second week of April. The peak in the fine fraction appears in the identical time period as in coarse particle filter samples (Fig 1A). Thus, the pollination season for birch is detectable in fine particulate matter even though the intact birch pollen grains are due to their size (~22.6 μm at the polar axis and 24 μm at the equatorial axis [57]) collected exclusively on the coarse particulate matter filters. The DNA found in the inhalable fine particle matter could stem from sub-pollen particles (SPPs) or plant debris. It is known that other birch plant parts besides pollen are potentially allergenic [25]. Together with the knowledge that during heavy rain or thunderstorms pollen can burst and release SPPs in great number, this issue becomes even more important for human health [58–60]. Therefore, if we inhale birch fragments within the fine particulate matter, we likely also inhale associated allergens, no matter if it is fragmented pollen or other plant debris in the samples.

Still, the chief allergenic risk during the main-pollination season is carried by the pollen grain themselves. The comparison of the DNA concentration in coarse and fine particulate matter from 2010-04-06 to the 2010-04-27 shows that for the ITS region the DNA copy number in coarse particulate matter is 64 times higher and for the *BP8* gene 51 times higher than in the corresponding fine particulate matter fraction. This difference emphasizes that the human immune system has to cope with both, the high number of pollen grains as well as the inhalable fraction of allergenic material.

Our results show not only differences in the DNA concentration between fine and coarse particulate matter but also between the quantified genomic regions. As shown in Fig 1A and 1B, during the pollination period in April the ITS region experienced a ~150–200 times higher DNA concentration than the *BP8* gene for fine and coarse particulate matter. The differences in the amount of detected copy numbers for the quantified regions, in the coarse as well as in the fine particle filter samples, is probably caused by the multi-copy character of the ITS region. The number of copies in the ITS region varies greatly among the plant kingdom, e.g., in higher plants the rDNA copy number is observed to vary between ten to more than ten thousand [61]. This huge range is also true for families, genera, and even for members of the same species [38]. The differences observed in this approach fit into this range. Despite their multi-copy character and the resulting uncertainties for DNA quantification, the ITS regions of nuclear ribosomal RNA provide, however, various advantages as a target region for qPCR compared to other loci. They are often preferred to single-copy genes as ITS sequences are available for many species in public databases [62] making it possible to easily design genus- or sometimes even species specific primer pairs. Although using a multi-copy area for measuring pollen quantities over-estimates the reality, it also has advantages, such as it allowing amplification of those samples containing only small quantities of pollen [62] or pollen fragments (as presumed to be in the fine particle fraction).

The decision to use a single-copy gene for more comprehensible qPCR measurements is thus based on the availability of target sequences in public nucleotide databases and the sensitivity of the primers.

### Comparison of DNA quantification, pollen count data, and simulation results

Unfortunately no traditional Burkard pollen sampling system was installed at the site where we measured the DNA concentration. For that reason no direct intercomparison of the results of both methods could be performed. Ideally the results of this first exploratory study should have been compared to pollen count measurements taken simultaneously and in close proximity to the air filter sampling device. As the best alternative we compare the measured DNA with measured pollen concentrations from the already existing Burkard trap at Löwenstein and with simulated pollen concentrations at both Mainz and Löwenstein.

**Fig 2:**
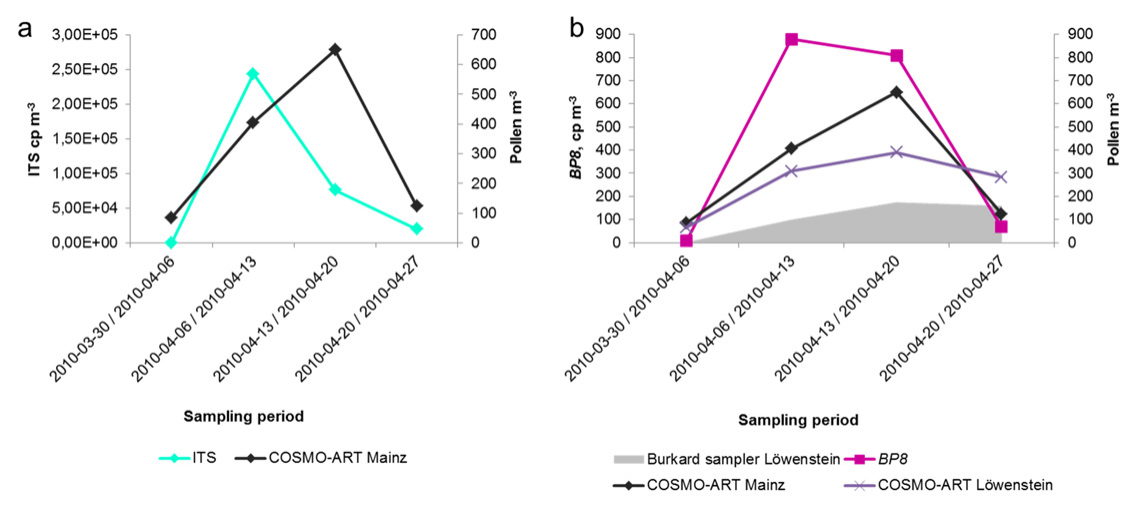
Comparison between qPCR measurements and COSMO-ART simulation. (a) DNA concentrations in copies m^-3^ (cp m^-3^) of coarse particle filter samples are given for multi-copy ITS region (cp m^-3^ air: primary vertical axis) and simulations of pollen concentrations from COSMO-ART (sum of pollen grains per m^3^ air: on secondary vertical axis) during the birch-specific main-pollination period in 2010 (2010-03-30 / 2010-04-27, x-axis) for Mainz (b) Quantification results for single-copy *BP8* gene (cp m^-3^ air: primary y axis) and pollen concentrations from COSMO-ART for Mainz and Löwenstein, as well as pollen concentrations averaged hourly in pollen m^-3^ sampled via Burkard traps (secondary y axis) are summed corresponding to the sampling interval of the appropriate filter sample(y-axis) during the birch-specific main-pollination period in 2010 (2010-03-30 / 2010-04-27, x-axis). Pollen counts were provided from nearest available pollen measurement station in Löwenstein, Baden-Wurttemberg about 130 kilometers south-east of Mainz.

For four weeks during the birch main pollen season in 2010, COSMO-ART was applied to simulate the dispersion of birch pollen grains in Germany based on the distribution of birch trees. The simulation results for Mainz were compared to the quantification of the coarse particle filter only, as on the coarse particle filters all intact birch pollen grains should be sampled. As illustrated in Fig 2A the results of the simulation predicted high pollen concentration in the first and second week of April 2010. The comparison with the ITS copy numbers strengthens this first peak. However, while the simulated pollen concentration increases also in the following week, the concentration of DNA copies in the ITS region decreases. This is different in the single-copy gene *BP8* as shown in Fig 2B. The DNA-copy concentration and simulated pollen distribution show similar patterns with peaks in the first two weeks of April. The temporal behavior over the four weeks was the same for *BP8* and the simulated pollen concentrations. In the first week the simulated pollen concentration was substantially higher than the quantified *BP8* gene copy number. In the following weeks the ratio between the simulated and measured pollen concentration was about a factor of two in week 2, a factor of 1.3 in week 3 and almost identical in week 4.

As *B*. *pendula* is a diploid plant [63] with tricelullar haploid pollen, and *BP8* is a single-copy gene that should therefore occur three times in each pollen grain. Under the assumption that the DNA recovered from aerosolized plant material is negligible as suggested by Rittenour et al 2012 [62] and—at least outside the pollen season- confirmed by the DNA quantification results which then are always lower than 1%, the *BP8* quantification results, divided by three should represent very roughly the actual birch pollen grain numbers. The true proportion of aerosolized plant material and pollen in air can be determined in a follow-up study. The differences between the observed quantification results and the model simulation are probably the result of the difficulty in estimating reliable pollen emission rates. Pollen emissions vary from tree to tree and from year to year [64].

The pollen trap data revealed that, during the birch-specific pre- and post-pollination period in Löwenstein, no pollen grains were detected (see Table 3), whereas the ITS and *BP8* primer pair quantified birch DNA in Mainz on almost all twelve analyzed filter samples. This difference is not surprising due to the spatial distance of 130 km between the two stations. As the pollen stations are assumed to represent only the pollen distribution over an area of about 20 km range [65], it is very likely that the air masses sampled in Löwenstein do not carry exactly the same amount of pollen number as if sampled in Mainz.

As shown in Fig 2B and Table 3, in the first week, the simulations in Löwenstein were substantially higher than the actual pollen counts ~3 times higher in the second week and 2 times higher in the last two weeks. In Mainz the simulated pollen concentration was compared to the quantified DNA. The *BP8* quantification was always higher than the simulated concentrations. Thus, in the comparison between all three methods, the lowest pollen grain numbers are found by the pollen trap method, and the highest with the DNA quantification method, with both methods experiencing the same detection limit of about one pollen per m^3^ air. One factor that could be responsible for the low pollen grain count numbers in the traps might be that they only analyze a small subsample of the 1 m^3^ air per day captured by the Burkard sampler. The high quantification results however, most likely originate from associated plant material during the pollination time. Although the relative proportions are not yet clear, this plant material is also of allergological interest.

To further strengthen the results we also performed statistical tests. To represent the qPCR data we chose *BP8*, as being a single-copy gene it is closest to a one-to-one relationship with pollen grain numbers as discussed above. The data sets from the *BP8* qPCR data in Mainz, the pollen trap in Löwenstein, and the COSMO-ART results at both sites are normally distributed, as tested by the Shapiro-Wilk-test, Table 4, [49]. We could therefore apply a Pearson linear regression to test on the one hand for correlation between qPCR and COSMO-ART in Mainz and on the other hand for a correlation between the pollen trap data and COSMO-ART in Löwenstein. With a Pearson-value of 0.92 between the COSMO-ART simulations and the Burkard pollen trap data in Löwenstein, it can clearly be shown that the simulation of the birch pollen season by COSMO-ART is well correlated with measurements (Table 4). The comparison of the qPCR results in Mainz with COSMO-ART also provide a correlation coefficient of 0.90 strengthening the observation that the *BP8* data reflects well the results directly simulated by COSMO-ART and indirectly–taking the comparison of COSMO-ART at both sites into account–the measured pollen trap data. As discussed above these exploratory results demonstrate that it is worth following-up on the DNA quantification approach. In a follow-up study, the proportion between pollen grain and plant material in particulate matter should be determined as well as the extraction efficiency for pollen grains. The results can then be used to correct the quantification results and produce reliable data to speedily and efficiently monitor pollen presence in the air.

**Table 4:** Statistical Analysis.

Data set	Shapiro-Wilk-Test	Pearson Correlation test,
	p-value	correlation coefficient *r*
*BP8*, Mainz	0.1	0.90
COSMO-ART, Mainz	0.44	
Burkard trap, Löwenstein	0.42	0.92
COSMO-ART, Löwenstein	0.41	

The Shapiro-Wilk-Test states, that data with a p-value higher than 0.05 is normally distributed.

## Conclusions

As increasing numbers of people are suffering from allergenic diseases mainly caused by airborne pollen, approaches from different scientific disciplines are being developed to better estimate the pollen and allergen load. In some aerosol studies allergens like Bet v 1 have been quantified directly [66–69]. Although, as discussed above, allergens occur not only in pollen but also in other plant material [25], at least for Bet v 1 there is no evidence yet for its presence outside the pollen season [68]. This direct approach will certainly be developed more within the coming years, but currently the diversity, allergenicity, and number of allergens in birch is not well understood [70].

In this exploratory study we, therefore, concentrated on DNA rather than the allergens themselves. We tested qPCR as a possible method to speedily quantify the pollen grain load in the air at genus or even species level and compared the qPCR of a single-copy gene and a multi-copy gene. We could show that the quantification of the ITS gene may increase the qPCR method sensitivity. However, the high variability in repeat numbers reduces the comparability to actual pollen numbers. Those are better reflected by the quantification of a single-copy gene such as *BP8*.

In the current study birch DNA was detected in the respirable fine fraction, albeit in smaller amounts than in the coarse fraction. The allergenic potential of the respirable fine fraction might be hazardous for human health as allergens within the pollen grain can be released when the pollen grain is fragmented into SPPs, e.g., after heavy rainfalls or thunderstorms [58–60,71]. The results of the model simulation and the single-copy based quantitative PCR were well correlated and open up a speedy and promising alternative to conventional pollen counting techniques. In addition, qPCR measurements allow identification on a species level, which is important, as not all species of a genus cause allergies [8]. Applied individually or in combination, both methods discussed here provide a promising approach to support or even replace conventional pollen monitoring systems in future. However, conventional microscopy analysis will also be needed in future to calibrate the alternative technologies until they evolve and become more economic options.
